# Supplementary material for: Integration of Cellular and Humoral Immune Responses as an Immunomonitoring Tool for SARS-CoV-2 Vaccination in Healthy and Fragile Subjects
Source: Viruses. 2023 May 30;15(6):1276. doi: 10.3390/v15061276 (PMC10305214; doi:10.3390/v15061276)
Supplement: Supplementary file 1 [file viruses-15-01276-s001.zip › viruses-2421676-supplementary.pdf]

viruses

Supplementary Material

1.1 Supplementary Tables

Supplementary Table S1. Humoral response in HCWs and association with clinical parameters

	IgG levels (AU/mL)	p-value*
**Autoimmune diseases**, median IgG_T1 (IQR)		
No	563.3 (360.0-917.0)	p = 0.645
Yes	724.4 (389.4-924.1)	
**Anti-inflammatory therapies during vaccination**, median IgG_T1 (IQR)		
No	572.8 (363.0-924.1)	p = 0.588
Yes	486.7 (280.3-1300.1)	
**Sex,** median IgG_T1 (IQR)		
Female	576.7 (376.0-948.4)	p = 0.375
Male	528.5 (334.1-863.6)	
**Autoimmune diseases**, median IgG_T2 (IQR)		
No	95.8 (70.0-179.4)	p = 0.402
Yes	117.4 (94.5-164.4)	
**Anti-inflammatory therapies during vaccination**, median IgG_T2 (IQR)		
No	99.2 (72.6-188.1)	p = 0.161
Yes	67.2 (9.9-168.2)	
**Anti-inflammatory therapies during immune response evaluation**, median IgG_T2 (IQR)		
No	101.4 (72.6-192.0)	p = 0.113
Yes	80.16 (42.4-168.2)	
**Sex,** median IgG_T2 (IQR)		
Female	110.1 (76.0-183.8)	p = 0.310
Male	91.5 (62.1-173.0)	
**Autoimmune diseases**, median IgG_T3 (IQR)		
No	735.7 (344.4-1754.2)	p = 0.952
Yes	837.9 (503.1-1129.0)	
**Anti-inflammatory therapies during vaccination**, median IgG_T3 (IQR)		
No	779.0 (351.70-1593.2)	p = 0.961
Yes	3521.0 (122.6-6919.4)	
**Sex,** median IgG_T3 (IQR)		
Female	779.0 (343.1-1482.4)	p = 0.828
Male	732.5 (351.7-1962.6)	

*Mann-Whitney test; Abbreviations: AU, arbitrary units; IQR, interquartile range; d, days after second vaccination dose; dB, days after the boost vaccination dose.

Supplementary Table S2. Humoral response in cancer patients and association with clinical parameters

	IgG levels (AU/mL)	p-value*
**Autoimmune diseases**, median IgG_T2 (IQR)		
No	50.2 (15.1-299.6)	p = 0.373
Yes	13.2 (13.2-13.2)	
**Anti-inflammatory therapies during vaccination**, median IgG_T2 (IQR)		
No	47.9 (15.1-299.6)	p = 0.604
Yes	32.8 (6.7-609.5)	
**Sex,** median IgG_T2 (IQR)		
Female	44.6 (11.5-164.3)	p = 0.310
Male	123.3 (13.2-665.7)	

*Mann-Whitney test; Abbreviations: AU, arbitrary units; IQR, interquartile range; d, days after second vaccination dose.

Supplementary Table S3. Cellular immune response in HCWs based on IgG_T2 tertiles

		Previous SARS-CoV2 infection	
	**All** (n=155)	**No** (n=133)	**Yes** (n=22)
**Ag1**, median IFN-γ IU/mL (IQR)			
I tertile	0.07 (0.03-0.29)	0.06 (0.03-0.29)	0.15 (0.08-0.30)
II-III tertiles	0.22 (0.08-0.48)	0.19 (0.07-0.39)	0.54 (0.28-0.88)
p-value	*0.001*	*0.005*	*0.061*
**Ag2**, median IFN-γ IU/mL (IQR)			
I tertile	0.09 (0.02-0.37)	0.07 (0.02-0.37)	0.18 (0.06-0.59)
II-III tertiles	0.28 (0.09-0.65)	0.23 (0.08-0.46)	1.04 (0.35-1.62)
p-value	*0.004*	*0.019*	*0.041*

Abbreviations: IU, International Units; IQR, interquartile range.

1.2 Supplementary Figures

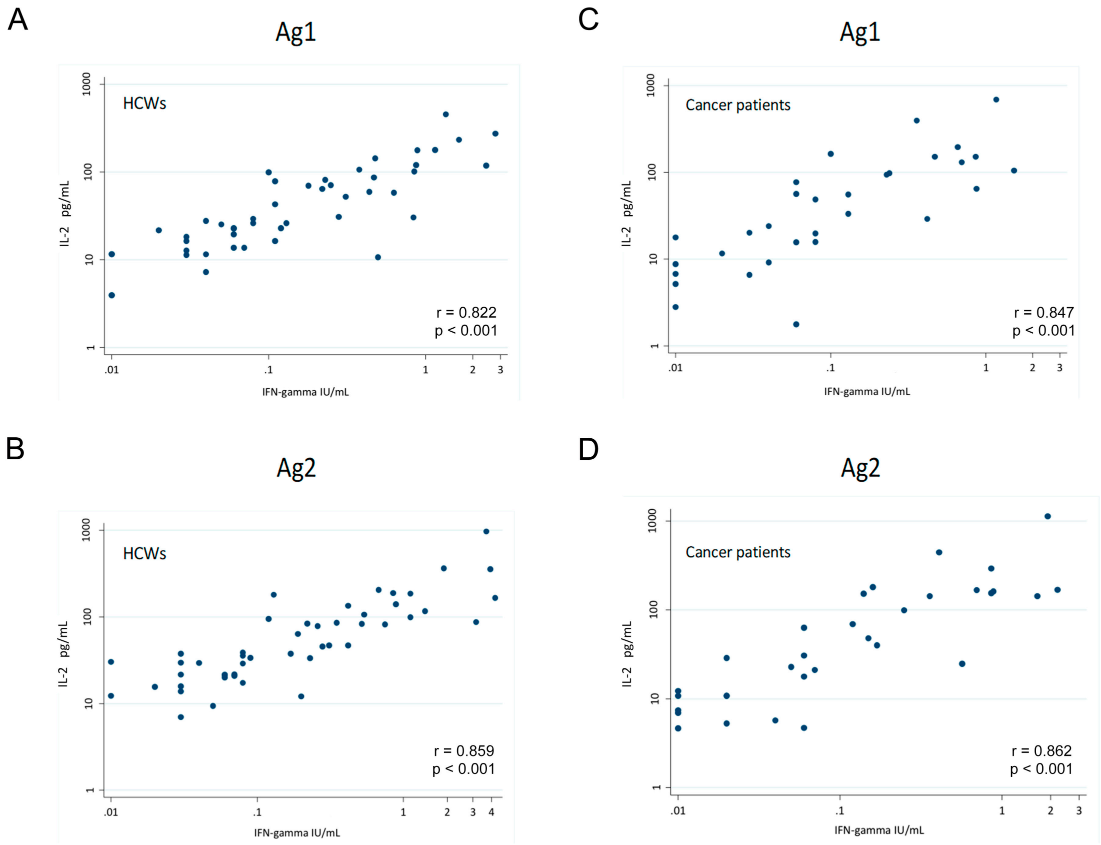

Supplementary figure S1. Correlation between IFN-γ and IL-2 levels at 120 days after the second (QF_T2) vaccination dose in response to Ag1 (A and C) and Ag2 (B and D) stimulation in the HCWs (A and B), and cancer patients cohort (C and D).

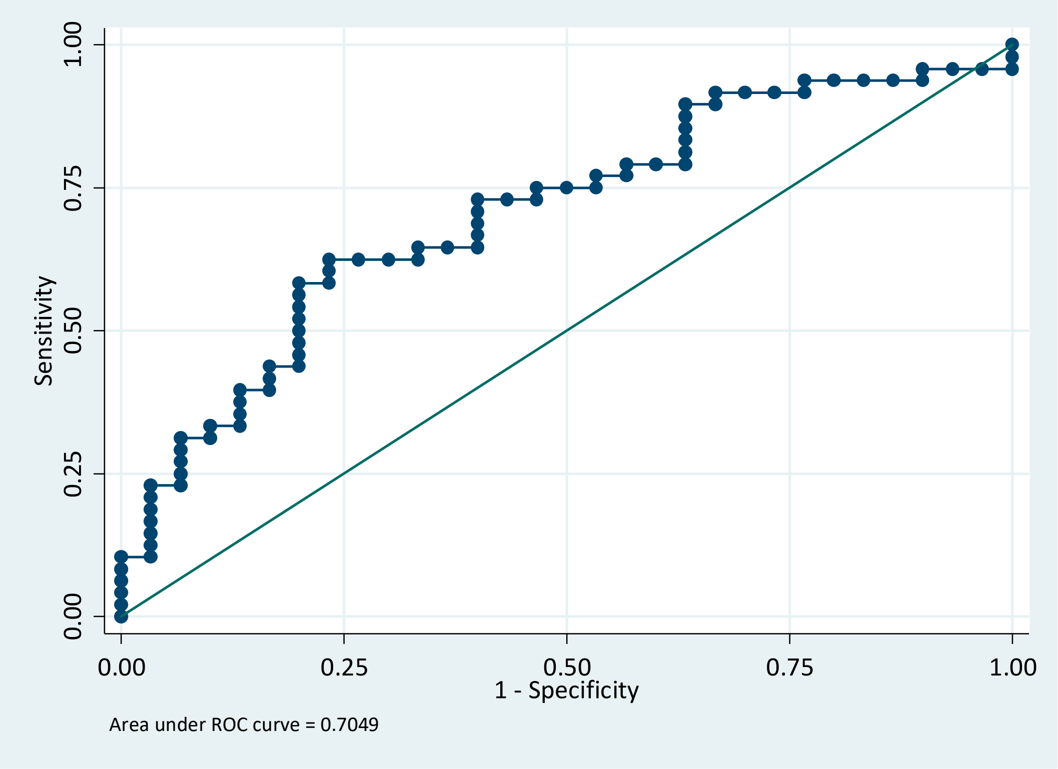

Supplementary figure S2. ROC curve analysis of anti-SARS-CoV-2 IgG levels at T2 showing sensitivity versus specificity for discrimination of HCWs resulted negative or positive for the presence of SARS-CoV-2-specific cellular immunity at T2.
